# Monthly Summary

**Published:** 1886-02

**Authors:** 


### Monthly Summary.

---

EXTRAORDINARY EFFECTS OF DRUGS.—At the recent meeting of the French Association for the advancement of Sciences, M. M. Bourru and Burot reported some remarkable experiments on two hystero-epileptics. With these subjects, an action is produced by any medicine wrapped in paper or even enclosed in a corked bottle and held at a distance of several centimeters from the body of the subject, without his knowledge and while he is in the waking state. At first are obtained the ordinary actions of irritation or inbibition; then very soon there is displayed a picture always the same for the same drug. This includes both psychical and physical phenomena, the last being the more prominent. Iodide of potash makes them sneeze and yawn. Opium puts them into a deep sleep, from which it is difficult to awaken them. Chloral produces a light sleep. Ethylic alcohol produces a happy intoxication, while methylic alcohol makes them furious. Ipecac causes vomiting. Scammony produces intestinal contractions. Cherry-laurel water produced, in one case, religious ecstasy followed by thoracic convulsions. Valerian gave with both subjects phenomena analogous to those which it develops in cats. Cantharides caused priapism and burning sensations in the urinary tract. Phosphorus caused trembling. Veratrine caused snuffling, itching of the nose and disturbance of vision.

Professor Duplong, who assisted at most of these experiments, affirmed the authenticity of the above report. He stated that the number of subjects capable of presenting the phenomena was very limited. Only two were found in Charcot's service, and none were found in the hospital at Grenoble. —*L' Union Medicale.*

